# Retraction Note: Eupafolin induces apoptosis and autophagy of breast cancer cells through PI3K/AKT, MAPKs and NF-κB signaling pathways

**DOI:** 10.1038/s41598-021-03369-7

**Published:** 2021-12-08

**Authors:** Jiahui Wei, Yu Ding, Xinmiao Liu, Qing Liu, Yiran Lu, Song He, Bao Yuan, Jiabao Zhang

**Affiliations:** 1 Department of Laboratory Animals, College of Animal Sciences, Jilin University, Changchun, 130062 Jilin People’s Republic of China; 2 The Second Clinical School of Medicine, Guangdong Provincial Hospital of Chinese Medicine, Guangdong Provincial Hospital of Chinese Medicine‑Zhuhai Hospital, Guangzhou University of Chinese Medicine, Guangzhou, 510120 Guangdong China

Retraction of: *Scientific Reports* 10.1038/s41598-021-00945-9, published online 02 November 2021

The Authors have retracted this Article.

After publication the Authors became aware of anomalies in two of the figures. Specifically:

- in Figure 1C, the middle panel in the left column for the invasion assay overlaps partially with the middle panel in the right column for the migration assay;
- in Figure 5D, the following four panels overlap partially: migration control, invasion control, migration 50uM and invasion 50uM.

All Authors agree with this retraction and its wording.

